# Sleeping Sickness Epidemics and Colonial Responses in East and Central Africa, 1900–1940

**DOI:** 10.1371/journal.pntd.0002772

**Published:** 2014-04-24

**Authors:** Daniel R. Headrick

**Affiliations:** Roosevelt University, Chicago, Illinois, United States of America; Institute of Tropical Medicine, Belgium

## Introduction

Human African trypanosomiasis, better known as sleeping sickness, nowadays ranks among the more neglected diseases in the countries of Africa where it is found. Though it still kills many people every year, it cannot compete for celebrity with such major killers as malaria and AIDS. Yet that was not always the case. A hundred years ago, sleeping sickness attracted considerable scientific research and political attention because of its importance to the conquest of sub-Saharan Africa by the European colonial powers. The goal of this paper is to describe the nature of the sleeping sickness epidemics that afflicted East and Central Africa in the early 20th century, the efforts made by European scientists to understand the disease and find means of controlling it, and the differences between the methods used by the British, Belgian, French, German, and Portuguese colonial authorities to combat it.

Sleeping sickness is a parasitic disease transmitted by tsetse flies. An infected person has joint pain, headaches, and a fever, then becomes drowsy. The infection also causes a swelling of the lymph nodes at the back of the neck. Once the pathogen crosses the blood-brain barrier and infects the central nervous system, the patient becomes lethargic or insane, then goes into a coma, and finally dies. There are two varieties of sleeping sickness, and they affect their victims very differently. One, caused by the protozoan *Trypanosoma brucei gambiense*, is a chronic disease that can persist for months or even years with occasional mild symptoms before it enters the central nervous system. The other, caused by *T. b. rhodesiense*, is acute and can cause death within three to 12 months of infection [1]–[3].

Africans were well aware that a closely related disease, animal trypanosomiasis, or nagana, caused fever and a progressive deterioration in the health of livestock, especially cattle. They knew it was transmitted by tsetse flies; in some areas they called them “canoe flies” because they were found near rivers or “elephant flies” because of their size. Cattle herders in East Africa avoided tsetse-infested areas or set fire to bush in order to clear areas of flies and of wart-hogs, bush-pigs, and other wild animals whose blood the flies fed on [4]–[6].

Sleeping sickness was endemic in many parts of Africa, with occasional epidemics, long before the colonial era. In the 14th century, the Arab historian Ibn Khaldun wrote that King Diata II of Mali had died of it. It was known to Europeans along the West African coast in the 18th century and in West Africa and the lower Congo in the 19th. According to John Ford, a specialist in the tsetse fly problem writing in the 1960s, Africans before the colonial era had established a rough equilibrium between two ecosystems, the human and domestic on the one hand, and the natural and wild on the other. Africans, whose ancestors had lived on that continent for hundreds of thousands (if not millions) of years, knew the habitats of tsetse flies and how to avoid them. This equilibrium was shattered by the invading Europeans, causing a series of ecological crises, including famines and epidemics of rinderpest, sleeping sickness, jiggers, and others [5], [7].

## The Epidemics

In the early 20th century, a series of epidemics across equatorial Africa brought the disease to the attention of the colonial powers. A particularly severe one broke out in 1901 in Uganda, especially along the shores and on the islands of Lake Victoria, a rich agricultural region with a high population density. By 1905 over 200,000 inhabitants, one-third of the population of the region, had died of the disease. It erupted in Sudan and German East Africa (now Tanzania) in 1904–1905 and in Northern Rhodesia and Nyasaland in 1908 [6], [8]–[12]. Hesketh Bell, the governor of Uganda, wrote in his diary, “The sleeping sickness problem overshadows everything else in my work here,” while Lord Edmond Fitzmaurice, British undersecretary of state for foreign affairs, called it “little less than a calamity for Tropical Africa” [2], [7], [10], [13]–[15].

Why sleeping sickness broke out in East Africa is not clear. Historian of tropical medicine Henry Scott attributed it to Henry Morton Stanley's expedition up the Congo River to rescue Emin Pasha in 1887 [6]. This hypothesis has been much disputed, however [16]. Sleeping sickness was probably endemic in the region and flared into epidemics as the expansion of colonial rule increased trade and migrations throughout Africa. This dramatic social change triggered outbreaks of sleeping sickness in several areas of East and Central Africa.

The outbreak of sleeping sickness was probably related to the epizootic of rinderpest that was introduced into Africa with the Italian invasion of Eritrea in 1889. This disease, to which African cattle had no resistance, killed between 90%–95% of them, along with many wild animals. By 1896, the epizootic had spread to the Cape of Good Hope, causing famine among herders throughout East Africa [1], [2], [7]. The British imperialist Lord Lugard commented that rinderpest “in some respects … has favoured our enterprise. Powerful and warlike as the pastoral tribes are, their pride has been humbled and our progress facilitated by this awful visitation. The advent of the white man had not else been so peaceful” [5].

A catastrophe for humans and their livestock was an opportunity for nature to reconquer the land they had vacated. As the cattle disappeared, bush and wild game reclaimed the abandoned pastures, and so did tsetse flies. Cattle herders, turning to hunting to survive, entered areas they had previously avoided. Increased trade and migrations spread the disease to West and Central Africa. British imperialists feared that the epidemic might spread down the Nile to Egypt and even to India, with disastrous consequences [5], [14], [17], [18].

Meanwhile, sleeping sickness was also epidemic in the French Congo and in Ubangi-Shari (now the Central African Republic). European visitors noticed a few victims in the late 1880s and increasing numbers along the Sangha and Ubangi rivers in the 1890s. In this swampy region, people relied on canoes for transportation. River people, fishermen, and canoeists were especially hard hit by the disease. In some places the infection rate reached 20% of the population. River towns were decimated; some disappeared entirely. The same was true across the Congo River in the northern parts of the neighboring colony of Congo Free State (later the Belgian Congo) [18], [19].

## The European Response

The participants in the Berlin Congress of 1884–1885 that partitioned Africa agreed that possession required effective occupation, and effective occupation meant introducing soldiers, traders, missionaries, and settlers in order to validate claims to territory. This triggered a burst of international rivalry between the colonial powers. The Portuguese occupied Angola and Mozambique for fear of losing them to Britain, as they had other parts of Africa they had once claimed but never really administered. France took over equatorial Africa north of the Congo River to preempt the Belgians, and Germany seized Southwest Africa (now Namibia) and German East Africa to preempt the British.

When the epidemic broke out in their colonies, the European colonial powers responded quickly, for several reasons. One motive was humanitarian. At the time, European humanitarianism consisted of a mixture of benevolent condescension and outright racism. The colonialists justified their intervention in Africa as saving hapless Africans from the diseases that plagued them.

There were more immediately practical considerations as well. Because pack animals could not survive in areas infested with tsetse flies, all transport, in an era before motor vehicles, depended on human porters or on canoes. Even before the epidemic, Africa—especially the equatorial zone—was thinly populated. Diseases exacerbated the manpower shortage, not only for transport but also for agricultural development, the collection of rubber, and other plans to exploit the colonies. From the colonists' point of view, sleeping sickness was an economic as well as a moral problem [13], [20]–[22].

Finally, there were scientific motivations. The turn of the century was the heyday of microbiology. The Frenchman Louis Pasteur had demonstrated the validity of the germ theory of disease and developed a vaccine for rabies, while Alphonse Laveran described the life cycle of the plasmodium of malaria. In Germany, Robert Koch had identified the pathogens of cholera, anthrax, and tuberculosis. In Britain, Patrick Manson and Ronald Ross identified the *Anopheles* mosquito as the vector of malaria. Throughout the Western world, studying diseases was an exciting challenge for a generation of microbiologists.

Among the advances relevant to this study was the discovery in 1895 by David Bruce of the British Army Medical Service of the pathogen of nagana (named *T. brucei* after him) in the blood of infected horses and cattle. In 1901 British colonial surgeon Robert Michael Forde observed “worms” in the blood of a sleeping sickness patient. The following year physician Joseph Everett Dutton identified them as the protozoa *T. gambiense* (now *T. b. gambiense*) [1]–[3], [15].

As European scientists working in the tropics identified specifically tropical diseases, their governments founded specialized schools of tropical medicine in the European metropoles to apply their findings to their colonies. Great Britain, with the most extensive colonial empire, led the way with two such schools. The Liverpool School of Tropical Medicine, founded in 1898 and directed by Ronald Ross, was associated with the merchant community of that city, while the London School of Hygiene and Tropical Medicine, founded the following year under Patrick Manson, was closely tied to the Colonial Office [16], [23], [24]. In 1900, the Pasteur Institute of Paris began establishing overseas branches. In 1902, the Portuguese government created a Colonial Hospital and School of Tropical Medicine in Lisbon to prepare military and naval personnel, merchants, missionaries, and government officials for work in the colonies [25]. These were followed by schools of tropical medicine in Marseille in 1905, Brussels in 1906, and Amsterdam in 1910 [4], [6], .

## The Scientific Missions

In response to the sleeping sickness epidemic, imperial governments sent specialists in tropical diseases to Africa to study the new scourge. Between 1901 and 1913, fifteen medical research missions (8 of them British) came to Africa to study sleeping sickness [28], [29]. In 1902 the Royal Society asked the London School of Tropical Medicine to dispatch a mission to Uganda. The leaders of the mission, parasitologist George C. Low and epidemiologist Cuthbert Christy, did little to advance knowledge of the disease. However, a third member of the mission, Aldo Castellani, a bacteriologist and student of Manson at the London School, established a small laboratory at Entebbe on Lake Victoria, where he identified several pathogens in the cerebrospinal fluid of sleeping sickness victims. For a while, it was not clear whether the pathogen that caused sleeping sickness was a bacteria, perhaps a variety of streptococcus called “hypnococcus,” or a protozoan, such as a trypanosome he called *T. ugandense* [1], [2], [15], [16], [30].

The following year, a second commission arrived in Entebbe led by David Bruce. Once in Entebbe, he identified the protozoan *T. gambiense* as the cause of the disease among the germs that Castellani had found in human blood. Members of his commission also showed that this parasite was transmitted by the tsetse fly, *Glossina palpalis*, that lived in the dense undergrowth along rivers and lake shores [1], [6], [10], [13], [15], [16], [28], [31], [32]. For years thereafter, a controversy raged between the supporters of Castellani and of Bruce over who discovered the pathogen of sleeping sickness [30], [33], [34].

The Portuguese government, eager to establish its bona fides as an imperial power, also sent missions to Africa. The first mission, sent in 1901 to Angola with a stopover on the island of Principe, included Annibal Bettencourt, director of the Royal Bacteriological Institute in Lisbon, Annibal Correia Mendes, director of the bacteriological laboratory in Luanda, and Ayres Kopke, director of the microbiological laboratory of the naval hospital in Lisbon and later director of the Lisbon School of Tropical Medicine. The purpose of this mission was not only scientific but also political; in the words of historian Isabel Amaral, it was “to display, for internal and external consumption, the scientific competence of the Portuguese mission, and to give a measure of the capacity of the Portuguese state to occupy and administer territories in Africa.” The Lisbon School of Tropical Medicine later sent other missions to Principe, Angola, Mozambique, Portuguese Guinea, and the Cape Verde Islands [3], [35]–[38].

In 1903, King Leopold II of Belgium asked the Liverpool School of Tropical Medicine to send a mission to the Congo Free State. After two years studying the disease, Christy, Dutton, and parasitologist John T. Todd recommended isolating the sick by imposing a cordon sanitaire around infected areas and establishing a series of *lazarets* or camps for sick Africans and those suspected of harboring trypanosomes [6], [15], [18], [22].

Until 1903, the German government showed less interest in sleeping sickness than in malaria, plague, and other diseases. Sleeping sickness was first reported in German East Africa in 1902. The following year the German Colonial Office took an interest in the matter and prepared to send an expedition [11], [22], [39]–.

Robert Koch, one of the most famous scientists of his time, led the mission to East Africa in 1906. Koch had previously led missions to South Africa, India, the Dutch East Indies, and Egypt. After stopovers in German East Africa, his mission set up camp on the Sese Islands in Lake Victoria, one of the most heavily infected areas in Africa [11], [39]–[41], [43], [44].

Koch's goal was to isolate the sick and experiment on them with a variety of arsenic-based compounds. The drug he found most effective and least toxic was atoxyl, or aminophenyl arsonic acid. First synthesized by the French chemist Antoine Béchamp in 1859, it proved effective in the short run but often left patients blind [39]–[41].

After Koch's departure, his associate Friedrich Karl Kleine described the developmental cycle of the trypanosome within the tsetse fly. He tried to set up isolation camps and forcibly move Africans away from tsetse-infested areas, but a lack of personnel and a fear of provoking an uprising prevented the implementation of these policies [39], [40], [42].

The French were the last of the major colonial powers to send missions to Africa to study sleeping sickness. That may be because their colonies in equatorial Africa—Gabon, Congo, Ubangi-Shari, and Chad—had few people and even fewer resources. In 1901–1902 parasitologist Émile Brumpt traveled across Africa from Djibouti to Brazzaville in the French Congo. He returned to the French Congo in 1903 to study tsetse flies and sleeping sickness [42], [45]. In 1907–1908 the Paris Geographical Society and the Pasteur Institute of Paris sent out a team led by Emile Roubaud, a medical entomologist, and two military doctors, Gustave Martin and Alexis Leboeuf. Thanks to the efforts of these and other scientists, the complex life cycle of the trypanosome in the digestive tract of the tsetse fly was unraveled in 1909 [4], [6], [46].

Finally, in 1910, pathologists John W. W. Stevens and Harold B. Fantham, working in Rhodesia, discovered a second and much more acute form of sleeping sickness that killed its victims within months, not years. This was caused by a different parasite, *T. rhodesiense*, usually transmitted by a different tsetse fly, *G. morsitans*, found in grassy areas rather than along riverbanks. *T. b. rhodesiense*, and perhaps also *T. b. gambiense*, had wild animals as reservoirs, but did not sicken their hosts. There was some overlap between the habitats of *G. morsitans* and *G. fuscipes*. Although both kinds of flies were found in some regions, within those regions, the two species were compartmentalized in particular ecological zones [3], [5], [6], [32], [33], [47].

Recently, T. Körner et al. have questioned the identification of the pathogen that caused the Ugandan epidemic of 1901. Instead of *T. b. gambiense*, they argued that the symptoms described at the time were more compatible with those of *T. b. rhodesiense* [1], [48]. If so, then there were not one but two simultaneous epidemics, one of *rhodesiense* in savanna regions and the other of *gambiense* in the rain forests.

News of the scientific findings spread quickly back to Europe, along with the returning scientists themselves. As Deborah Neill has shown, one important outcome was international cooperation among scientists and physicians [13]. German physicians visited the laboratory of Castellani and Bruce in Entebbe. At a major conference on sleeping sickness organized in 1907–1908 in London by Patrick Manson, delegates from Britain, France, Germany, and Portugal discussed not only the recent findings but also drugs and prophylactic measures. The Colonial Office and the Royal Society created a Sleeping Sickness Bureau to collate and publicize information [16], [28], [29], [39]. Ronald Ross of the Liverpool School of Tropical Medicine corresponded with the Frenchman Dr. Gustave Martin and with the French Geographical Society and the Pasteur Institute. Dr. Ayres Kopke maintained relations with Patrick Manson. In German East Africa, Dr. Oskar Feldman corresponded with Dr. Cuthbert Christy. On a diplomatic level, Germany and Great Britain agreed in 1908 to prevent infected Africans from crossing borders, and in 1911 they signed a cooperative agreement to combat sleeping sickness in West Africa.

The First World War interrupted these efforts. The return of peace in 1918 did not lead to a renewal. Germany and France remained hostile for years after the war. Yet, slowly, cooperation returned. In 1923 the medical authorities of Angola organized a Congress on Tropical Medicine in Luanda with the goal of establishing a scientific alliance among African colonies so that tropical diseases could be studied in their natural habitat. In 1924 the League of Nations convened an International Expert Committee on Tuberculosis and Sleeping Sickness and sent seven experts to Entebbe. In 1927, Britain hosted an International Conference on Sleeping Sickness in London and the following year the League of Nations Health Organization sponsored a second conference in Paris [6], [25], [29], [36], [44]. These conferences helped disseminate information about drugs and preventive measures, such as establishing an international cordon sanitaire to prevent the spread of the disease.

## Cure and Prevention

So many missions and so many conferences had less impact on the course of the epidemics than the scientists had hoped. One reason is that the protozoan that caused the disease outsmarted the scientists, for it changed its protein coat frequently, thereby thwarting efforts to design a vaccine that could help the human body develop the antigens necessary to fight the infection. Nor was there a natural drug, such as quinine for malaria, that could prevent infection. The methods used to prevent infection and to cure the infected were drastic, and the results were limited. Yet they laid the basis for the public health campaigns of later years.

In the early years of the epidemic, atoxyl was tested against sleeping sickness by Robert Koch in Uganda and by Ayres Kopke in Lisbon. Kopke, representing Portugal, reported on its effectiveness at the International Conference on Sleeping Sickness in London and at the International Congress of Hygiene and Demography in Berlin in 1907. Despite its name, which means “nontoxic,” atoxyl was dangerous, for it caused partial or total blindness in up to 20% of patients. Later research showed that it had a chemotherapeutic index close to one, meaning that the minimum dose needed to rid the patient of trypanosomes was almost the same as the maximum dose that patients could tolerate without being poisoned to death. Yet it was cheap, stable in the tropics, and easy to inject; hence, it remained in use long after other, better drugs became available.

Because of atoxyl's shortcomings, researchers in Germany looked for drugs that would be both more effective and less dangerous. Germany, at the time, had the most advanced chemical and pharmaceutical industry in the world. The term chemotherapy was coined by the German pharmacologist and immunologist Paul Ehrlich to refer to the creation of a chemical that would attack a specific pathogen, a *Zauberkugel*, or “magic bullet,” as Ehrlich called it. Unlike natural drugs like quinine or poisons like mercury or arsenic that had long been used by doctors against a variety of diseases, chemotherapeutic drugs were synthesized in a laboratory. Research focused first on testing synthetic dyes in order to identify the one that would attach itself to a particular pathogen, then on attaching that dye to a toxin that would kill that pathogen. Closely associated with the chemical manufacturer Hoechst, Ehrlich and his assistant Dr. Sahachiro Hata tested hundreds of combinations of chemicals against trypanosomes. Some, like Trypan Red, proved effective in animals but too toxic for humans. In the process, they synthesized Salvarsan, the first effective drug against syphilis. It became, for a while, the most widely prescribed drug in the world, for which Ehrlich received the Nobel Prize [13], [35], [37], [38], [49]–[51].

Meanwhile in 1916, at another German chemical firm, Bayer, Oskar Dressel, Richard Kothe, and Wilhelm Roehl, Ehrlich's former assistant, developed 205 Bayer, which the company patriotically dubbed “Germanin.” After the war, it offered to reveal the secret formula in exchange for the return of Germany's colonies, but France and Great Britain turned down the offer. Instead, the French pharmacologist Ernest Fourneau at the Pasteur Institute reverse-engineered the drug on the basis of patents that Bayer had taken out and named it 309 Fourneau [52], [53]. It was commercialized by the Rhône-Poulenc pharmaceutical company under the name Moranyl. With a chemotherapeutic index of one to sixty or more, this drug was much safer than atoxyl and could even be given to healthy people as a prophylactic. Fourneau also created Orsanine, another drug that was effective but very expensive. Meanwhile, trypanosomes were beginning to show signs of resistance to atoxyl and other drugs. In this area as in so many others, the arms race between pathogens and pharmacologists had already begun [7], [19], [49], [54], [55].

While effective against the first stage of sleeping sickness, none of these drugs could reverse the course of the disease once the trypanosomes had penetrated the blood-brain barrier. Another drug called tryparsamide, which was effective in patients with second-stage sleeping sickness and had a chemotherapeutic index of one to two, was developed by Michael Heidelberger and Walter Jacobs, two Americans working at the Rockefeller Institute in New York. However, it was expensive. Whereas atoxyl cost an average of 6.9 French francs per patient, tryparsamide cost 44 francs and Orsanine, 55 francs [2], [19], [49], [56].

## The British Environmental Approach

Colonial officials developed different approaches to this disease. As Michael Worboys pointed out, one was environmental, namely, separating humans from tsetse flies; the other was medical, namely, attacking the trypanosomes in order to cure the sick and prevent the transmission of the pathogens to the healthy. The colonial powers used both approaches, but in very different proportions. Let us examine these approaches and then ask what explains the differences between them [31].

The environmental approach that the British adopted in East Africa has been the subject of works by Kirk Hoppe and Harvey Soff, among others. It originated with Dr. David Bruce. He recommended first separating Africans from tsetse flies and then eliminating the flies by destroying their breeding places and the wildlife he thought carried the pathogen. Hesketh Bell, appointed governor of Uganda in 1906, decided on his own initiative to implement this radical idea. He ordered all Africans to move to fly-free areas two miles or more away from the lakeshore and the islands of Lake Victoria and forbade fishing and the sale or possession of fish. Hunting and gathering firewood in the infected areas were forbidden. Kampala Port remained accessible to Africans, but only to those who had been medically examined and registered. In Bell's words, “We must withdraw from the insects the source of their infection. The whole country must be depopulated. There seems to me to be no other course than to remove *everyone* from reach of the fly for an indefinite period.” He also established camps where the sick could be treated [9], [15], [16], [57].

Needless to say, this caused great hardship among Ugandans who lived and farmed near the lake and among the many fishermen who provided one of the few sources of protein for the population. Some resisted, others sneaked back to hunt or fish. As the farmers left, bush invaded their abandoned fields and so did tsetse flies. Yet the results were remarkable. Whereas in the years 1900–1904, at the height of the epidemic, 200,000 out of the 300,000 inhabitants of the infected areas died of sleeping sickness, between 1905 and 1909, fewer than 25,000 died. By 1910 the epidemic had tapered off and Africans began returning to their former homes [9], [10], [22], [31], [57].

One aspect of the fight against sleeping sickness was clearing bush, especially in the vicinity of streams and lakes and near human habitations. In Uganda, the British made Africans plant citronella grasses in the cleared areas in the belief that they would repel tsetse flies. But once the population had been evacuated, such a measure was bound to be short-lived, as the bush reclaimed its native habitat.

Another environmental method was game control. At the time, scientists had no evidence that the trypanosomes that afflicted humans could live in wild animals without infecting them, yet some of them suspected as much. This caused a major controversy. David Bruce lobbied for game eradication. Like Bruce, Robert Koch believed that wild animals were reservoirs of sleeping sickness, singling out crocodiles as the guilty party. The Treaty on the Combat of Sleeping Sickness in East Africa that Great Britain and Germany signed in October 1908 called for an all-out war on crocodiles. This view, however, ran into fierce opposition from British big-game hunters, many of whom were governors and highly placed officials. In the end, the big-game hunters won, and game eradication was never carried out effectively [5], [10], [15], [16], [40], [58].

## Tanganyika

The medical establishment of German East Africa had only begun to stem the sleeping sickness epidemic when World War I put a stop to their efforts. As Mari Webel has shown, they were hindered by a lack of medical personnel. In the kingdom of Kiziba on the western shore of Lake Victoria, one physician, Dr. Robert Kudicke, attempted to implement the policies recommended by Robert Koch: identifying the sick and treating them with atoxyl. To identify potential patients, he employed “gland-feelers,” young men associated with the court of the king whom he trained and paid to palpate the lymph glands of villagers and bring those with swollen glands to a camp at Kigarama. The system, established in 1907, had treated 581 patients by late May 1908, a small but significant number in the midst of an epidemic. By then, the gland-feelers began encountering resistance, as villagers fled to avoid their authority and feared the treatment they would receive in camp. As people became convinced that the atoxyl regimen was more harmful than effective, the king withdrew his support and Kudicke stopped employing gland-feelers [59]. During the war, fighting, labor conscription, and requisitions of crops and livestock caused farmers to abandon their fields, which reverted to bush, the preferred habitats of tsetse flies. After the rinderpest epidemic abated, wildlife recovered more quickly than domesticated cattle, and with the wildlife came the tsetse flies [45].

The British colonial administrators, who took over the colony from Germany and renamed it Tanganyika, therefore inherited a serious sleeping sickness problem. Like their counterparts in Uganda, they pursued a tsetse-focused approach to the disease. In 1919, they appointed Charles Swynnerton, a rancher and self-taught entomologist, as director of game preservation. Two years later, they put him in charge of tsetse control. Swynnerton had observed Zulu herders in Mozambique set fires every year to rid the countryside of ticks and tsetse flies and he was determined to do the same in Tanganyika. In northern Tanganyika, especially near Lake Victoria, Swynnerton made Africans burn all vegetation that might harbor the flies and thereby create “fly barriers” around human habitations. As director of the Tsetse Research Department, he had a staff of 21 Europeans and 122 Africans. In 1926–1928, 12,000 square miles of tsetse-infested land were evacuated and their inhabitants relocated to areas deemed free of flies. Travel was strictly controlled and pedestrians and vehicles were searched for flies. Animals that might harbor trypanosomes were hunted down. The areas that were thereby depopulated remained so for a long time thereafter; many became the wildlife reserves for which Tanzania is famous today [11], [14], [28], [29], [47], [57], [60], [61].

## The Belgian Medical Approach

In the Congo Free State, as the intensified Belgian presence caused increased movements of people and their pathogens, sleeping sickness spread along the rivers.

In response to the epidemic, the Belgians adopted a policy that differed radically from that used by the British in East Africa. As Maryinez Lyons has shown, instead of attempting to separate humans from flies, they set out to destroy the trypanosomes in sick Africans, thereby preventing their transmission to healthy ones [18]. Following the recommendations of the mission by the Liverpool School of Tropical Medicine in 1903–1905, the colonial government instituted stringent police measures. It imposed a cordon sanitaire around fly-infested areas and controlled the movement of people, requiring medical passports for travelers. It opened camps for the sick, staffed by Catholic nuns. Africans were diagnosed by palpating their neck glands. Those suspected of being infected were herded into these camps, where they were isolated from outsiders and injected with atoxyl. These camps proved to be unpopular because of the painful treatment, poor conditions, lack of food, and permanent separation of patients from their families. To prevent the sick from escaping, they had to be guarded by soldiers [13], [18].

The epidemic peaked during World War I, in part because the authorities forced Africans to collect rubber from *Landolphia* vines in tsetse-infested areas; those who did not meet their quota were conscripted into labor brigades or as porters, causing the disease to spread even further. Infection rates in different provinces ranged from 10%–29% of the population, just as doctors—many of them Italians—were drafted to serve with the armies in Europe [18].

After several years, the medical authorities shifted to decentralized ambulatory care. The medical corps sent itinerant teams to examine villagers; by the 1930s, they were examining 3 million people, or 70% of the population, every year. They also opened rural clinics, hospitals, and injection centers, especially in the western provinces. The campaign against endemic diseases was conducted by the public health service as well as by a specialized anti-sleeping sickness service and by Catholic and Protestant missions [18].

Overall, the campaign was effective; by the 1940s there were fewer new cases than in the past. The Belgian Congo won praise from Europeans for offering the most effective and comprehensive medical care in any European colony, and the Belgians touted their health care system as a proof of their civilizing mission. For Africans, however, it meant living in a police state, with a health care system that only overcame an epidemic that European colonial rule had exacerbated in the first place [2], [5], [18], [22].

## The French Approach

French Equatorial Africa (*Afrique Équatoriale Française* or AEF) was poor in resources and thinly populated, even before epidemics of sleeping sickness and other diseases decimated the population. The French first noted sleeping sickness in the late 19th century at the mouth of the Congo River. As they moved inland, their soldiers, canoeists, porters, and houseboys spread the disease up the rivers from the Atlantic coast to Chad. Populated areas of Ubangi-Shari and Chad were soon affected. The Martin Leboeuf Roubaud mission in 1906–1908 advocated isolating the sick, administering high doses of atoxyl, and clearing the undergrowth that harbored tsetse flies. But resources were limited and little was done. In 1908–1909, a new governor, Martial Merlin, made sleeping sickness eradication a priority and founded the Pasteur Institute of Brazzaville. A camp was set for up to 120 patients, but they were poorly housed and fed and tended to wander off, spreading the disease. Other measures, such as clearing underbrush near settlements, limiting population movements, and requiring health passports for passengers on river steamers, were rarely enforced [19].

What changed the situation was the energy and initiative of one man, Dr. Eugène Jamot, director of the Pasteur Institute in Brazzaville [19], [62]. In 1917, Jamot devised a system of mobile medical teams consisting of a French military doctor, seven African male nurses trained at the Pasteur Institute in Brazzaville, two white corporals, several African soldiers, and a large number of porters to carry all their equipment. His goal was neither to treat the sick nor to eradicate the flies, but to kill the trypanosomes in the entire population, thereby reducing the risk of infecting the healthy. Teams went from village to village. In each village, the inhabitants were required (often at gunpoint) to submit to examination. The doctor and nurses palpated their neck glands for telltale swelling and examined their blood and lymph under a microscope. In rare cases, they also performed spinal taps to check the cerebrospinal fluid of those suspected of harboring trypanosomes. Patients with second-stage sleeping sickness were usually ignored because spinal taps were difficult and risky, and besides, there were no drugs that could help. In some infected areas, the teams administered atoxyl to everyone; even after much better drugs became available and evidence mounted that trypanosomes were becoming atoxyl-resistant, the French continued using it because of its low cost. By this assembly-line method, they could examine and treat up to a hundred cases a day. For lack of funds, however, follow-up visits were impossible, and the teams would not return for months.

Jamot first tried his method in the colony of Ubangi-Shari, north of the French Congo. In 1917–1919, his team visited almost every village, examined 89,643 people, and found 5,347 cases of sleeping sickness. Yet going on circuit was exhausting for doctors and nurses and for the porters who had to carry all their equipment on their heads. Traveling was difficult in all seasons, and almost impossible in the rainy season. Patients were supposed to be checked every few months to see if they were cured and, if not, to receive further injections of atoxyl, but many fled into the bush at the approach of the medical teams [4], [19], [47], [63].

In 1922 Jamot was transferred to Cameroon, where the French were eager to show that they did not neglect their African subjects, as the German press claimed. In fact, before World War I, the German administrators of Cameroon had done very little to control the epidemic for lack of physicians and of funds. Though they obtained subsidies from the French Compagnie Forestière Sangha-Ougangui, they blamed the spread of sleeping sickness on the natives of the neighboring French Congo. After the war, the German press argued that their contribution to pharmacology proved their concern for Africans was greater than that of the French [39], [41], [64].

Once posted to Cameroon, Jamot set up a permanent system of mobile teams that visited villages and treated the sick. Each team carried eight to 14 microscopes with which the nurses could detect trypanosomes in blood. In 1928, these teams examined 663,971 Africans, of whom 115,354 (or 17%) were infected; in some areas, half the inhabitants were infected and were injected with atoxl or tryparsamide. To break the epidemic required several visits and injections over a five-year period [65], [66].

Meanwhile in AEF, following Jamot's example, the colonial administration created a special organization, the *Service de la prophylaxie de la trypanosomiase*, which became operational in 1927. Gradually, as funds became available, the number of doctors rose from seven in 1928 to 29 in 1934 and the number of nurses from 105 to 243. For the sick, there were 60 treatment camps. Though in their single-minded focus on sleeping sickness the French colonial administrators neglected all other diseases, they finally overcome the epidemic by the late 1930s, and the population, long stagnant, began to grow [19].

## The Portuguese Approach

Portugal was a very much poorer nation than Great Britain, France, or Belgium, yet it possessed an enormous colonial empire in Africa, many parts of which were afflicted with sleeping sickness. From the beginning, as we saw earlier, it engaged in the struggle against the disease through research missions and participation in international conferences. On the ground, it registered one major triumph against the disease in a very small part of its colonial empire: Principe.

Principe is an island of 52 square miles (half again as large as Manhattan) in the Bight of Benin. Since the mid-19th century, its main product was cacao beans. Despite the immigration of workers from Gabon, Congo, and Angola, its population plummeted from 3,000 in the mid-19th century to 800 in 1900 and to 350 in 1907. Six hundred workers imported from Angola in 1894 died within five years. The drop in population caused a decline in the cacao crop and aroused the Portuguese government to action.

In 1901, the first Portuguese sleeping sickness mission stopped there on its way to Angola. The second mission, sent in 1907, did a thorough study of the disease on the island and recommended a series of measures to eradicate it. A third mission, led by Bernardo Francisco Bruto da Costa, was sent to Principe in 1911 with legal powers to enforce these recommendations. Convicts were imported from nearby colonies to clear undergrowth near human habitation, drain swamps, and fell trees. Workers wearing black cloths, to which tsetse were attracted, went around catching and killing the flies. Wild pigs, civet cats, and monkeys were hunted and killed, as were stray dogs. All the inhabitants were examined and injected with atoxyl, and the sick were segregated in special camps. Villages in infested areas were moved and the inhabitants were monitored. The results were astonishing. The proportion of inhabitants with trypanosomes in their blood dropped from 26% in 1907 to 0.64% in 1914. From then until the 1950s, Principe was effectively free of tsetse flies and of sleeping sickness [30], [36]–[38], [67], [68]. But, as British colonial undersecretary William Ormsby-Gore remarked after visiting Principe, “It is one thing to deal with an island and altogether another proposition to deal with a continent” [69].

The Portuguese success in eliminating sleeping sickness from Principe showed what could be done by applying, simultaneously and in a concentrated dose, all the methods used by the British, the Belgians, and the French, using a lot of forced labor in a very small area. In its larger colonies, Angola and Mozambique, neither the Portuguese government nor the colonial authorities had the money or manpower to stem the epidemic.

## Conclusion

All the European colonies in Central and East Africa suffered from simultaneous epidemics of sleeping sickness. Yet the responses of the colonial authorities differed radically. To what can we attribute the differences?

Historians of comparative imperialism have often noted the differing styles of colonial rule. The British are said to have preferred indirect rule, using traditional native leaders whenever possible. The French ruled directly, employing French soldiers or civil servants, even at the local level. In French Africa, medicine was largely in the hands of army doctors who were posted to the colonies so they would be available in the event of a war in Europe. Belgian colonialism was paternalistic and heavily influenced by the Catholic Church. And Portugal was mainly concerned with gaining international recognition in order to protect its colonies from other predatory colonial powers.

These generalizations only go so far, however. To understand the differences, we need to delve into the scientific culture of the colonizing nations. The French, following in the footsteps of Louis Pasteur and Alphonse Laveran, tended to focus on identifying and eradicating the pathogens rather than on the vectors of diseases. The British, inspired by Ronald Ross, Patrick Manson, and David Bruce, concentrated on the vectors. The Portuguese approach to sleeping sickness was motivated by political as well as scientific considerations. And the Germans excelled in chemistry and pharmacology [70].

Nor should we ignore the importance of individuals. In Uganda, Hesketh Bell imposed his solution—removing people from tsetse-infested areas—upon a recalcitrant population. In Tanganyika, Charles Swynnerton attempted to destroy the habitat of the flies. In French Equatorial Africa and Cameroon, Eugène Jamot, a physician trained in the Pasteurian tradition, attacked the trypanosomes rather than the flies.

Finally, ecological factors also played a role. Uganda and Tanzania are largely open savanna, except for lakeshores and riverbanks where vegetation grows more thickly. Much of AEF and the Belgian Congo, in contrast, consists of rain forests and wetlands similar to Amazonia; with a very small population thinly spread over very large areas, the idea of clearing bush and draining swamps was completely out of the question.

In the end, the success of the colonial medical authorities in fighting sleeping sickness before 1940 reversed the decline in the health of Africans in the preceding 50 years. By the 1930s, the number of cases gradually diminished. After independence, population growth, civil disorder, and political problems interrupted this downward trend and provoked a new epidemic. In 2008, according to the World Health Organization, between 50,000 and 70,000 persons were infected, while Doctors Without Borders estimated the number of infected persons at 300,000 [2], [39]. Accurate statistics are hard to come by, especially because the most tsetse-infested areas, such as the eastern Congo and the Central African Republic, are also places of endemic warfare and banditry, in which health care is largely absent. Elsewhere, sleeping sickness is currently under control; at least until a new epidemic breaks out, taking the health services by surprise. Meanwhile, there are more important health issues for the world to worry about, such as malaria, AIDS, and malnutrition, so sleeping sickness has become a footnote in history.
